# The prevalence of intraspinal anomalies in infantile and juvenile patients with “presumed idiopathic” scoliosis: a MRI-based analysis of 504 patients

**DOI:** 10.1186/s12891-016-1026-7

**Published:** 2016-04-27

**Authors:** Wen Zhang, Shifu Sha, Leilei Xu, Zhen Liu, Yong Qiu, Zezhang Zhu

**Affiliations:** Department of Spine Surgery, Drum Tower Hospital of Nanjing University Medical School, Zhongshan Road No. 321, Nanjing, 210008 China

**Keywords:** Infantile idiopathic scoliosis, Juvenile idiopathic scoliosis, Magnetic resonance imaging evaluation, Neural axis abnormalities, Prevalence

## Abstract

**Background:**

Though several studies have reported the incidence of intraspinal neural axis abnormalities in infantile and juvenile “presumed idiopathic” scoliosis, there has been a varying prevalence ranging from 11.1 to 26.0 % based on a limited sample size. Therefore, such inconclusive findings have resulted in some questions on the MRI-associated role in the management of these patients. We aimed to investigate the prevalence and distribution of intraspinal anomalies in the infantile and juvenile patients with “presumed idiopathic” scoliosis and to explore the radiographic and clinical indicators with large sample size.

**Methods:**

A total of 504 infantile and juvenile patients diagnosed with “presumed idiopathic” scoliosis were examined for potentially-existing neural axis abnormalities by MRI. Patients were grouped into two cohorts according to the presence of neural axis abnormalities. Radiographic parameters including curve magnitude, curve pattern, location of apex, degree of thoracic kyphosis, and span of curve were recorded and compared between the two groups. The prevalence of the neural abnormalities between the infantile-age group and juvenile-age group was also compared. The student *t* test was used to evaluate the differences of continuous variables and the *chi-square* test was used to evaluate the difference of categorical variables. Fisher exact test was applied to detect the difference of the rate of intraspinal anomalies between the “infantile idiopathic scoliosis” and “juvenile idiopathic scoliosis” group.

**Results:**

Involving the spinal cord, 94 patients (18.7 %) were found to have a neural abnormality: Arnold-Chiari malformation alone in 43 patients, Arnold-Chiari malformation combined with syringomyelia in 18 patients, isolated syringomyelia in 13 patients, diastematomyelia in six patients, tethered cord combined with diastematomyelia in six patients, tethered cord alone in four patients, and other uncommon intraspinal abnormalities in the remaining four patients. Totally Arnold-Chiari malformation with or without syringomyelia accounted for 64.8 % (61/94) among all these abnormalities. Male gender, left thoracic curve and right lumbar curve were found to be significantly associated with the presence of neural axis abnormalities on MRI.

**Conclusions:**

The incidence of neural axis abnormalities in the presumed IIS and JIS was 18.7 %. Thus a routine MRI evaluation appears warranted for those “presumed idiopathic” scoliosis patients if aged less than 10 years, being male or having left thoracic or right lumbar curve.

## Background

As a common three-dimensional spinal deformity, idiopathic scoliosis (IS) can be categorized into infantile idiopathic scoliosis (IIS), juvenile idiopathic scoliosis (JIS) and adolescent idiopathic scoliosis (AIS) according to the age at onset. Although the IIS and JIS account for a small part of IS, they were reported to be more frequently associated with intraspinal abnormalities [1], which, if remain undetected, could add to the risk of neurological sequelae following surgical correction of scoliosis.

Over the past two decades, although the full spinal MRI has been recommended to detect the neuro-axis abnormalities in patients with presumed IIS and JIS, the incidence of intraspinal neuro-axis abnormalities by MRI still remains obscure [1–8]. Meanwhile, some questions have been raised that the full spinal MRI seemed over-used in presumed IIS and JIS patients considering the relative lower incidence of intraspinal neuro-axis abnormalities [8, 9]. Additionally, most clinical and radiological indicators for the use of MRI in literature have been applied to AIS patients [10, 11], while they were still unconfirmed or even neglected in presumed IIS and JIS patients. It is supposed that these limitations could be attributed to the relatively small sample sizes and the heterogeneity of the age criteria of IIS and JIS in previous studies [1–8]. To the best of our knowledge, there has been no study conducted with a large sample size to determine the incidence of intraspinal anomalies in patients with presumed IIS and JIS. Therefore, a MRI-based study with a large sample size should be performed for a more conclusive prevalence of intraspinal neuro-axis abnormalities and will undoubtedly shed light on the evidence for applying full spinal MRI screening to the presumed IIS and JIS. In the current study, the records of patients with “presumed idiopathic” scoliosis aged less than 10 years were screened. A cohort of 504 patients were selected for inclusion that met the criteria to investigate the prevalence and distribution of intraspinal neuro-axis anomalies and to explore the radiographic and clinical indicators for such anomalies.

## Methods

The Drum Tower Hospital’s review board approved this retrospective study and waived the requirement for written informed consent.

### Subjects

The database of a single spinal deformity center was retrospectively reviewed to identify all patients with a diagnosis of IIS or JIS between January 2003 and October 2013. According to the Scoliosis Research Society, IIS is defined as the age at onset younger than 3 years and JIS is defined as the age at onset ranged from 4 years to 10 years.

The inclusion criteria were as follows: (1). aged ≤10 years at diagnosis; (2). with a normal neurologic finding on history and physical examination; (3). with an initial primary curve magnitude > 20°; (4). with total spine MRI screened from the skull to the coccyx. Patients with neuromuscular conditions, syndromes, congenital scoliosis or any structural deformities of spine were excluded. Neurologic examination was performed for all patients by a spinal orthopedic surgeon at the first visit, including evaluation of the motor, sensory, and reflex function of the upper and lower extremities as well as an evaluation of the abdominal reflex.

### Clinical and radiographic assessments

The total spine MRI was carried out with a 1.5 Tesla Philips Magnetom system (Philips Medical Systems, Netherland) for detection of potential neural axis abnormalities, including Arnold-Chiari malformation, syringomyelia, diastematomyelia, tethered cord and neoplasm, which were reviewed by an attending spine surgeon and an experienced radiologist respectively. The diagnosis of an Arnold-Chiari malformation was made when the herniation of the tonsil was below the foramen magnum by 5 mm or more [12], and the diagnostic criteria for tethered cord was determined by a conus medullaris below the L1-L2 space and/or a filum terminale >2 mm in thickness [13]. Diastematomyelia is defined as a form of spinal dysraphism characterized by a division of spinal cord or caula quina [14]. Syringomyelia is a term that delineates conditions of abnormal fluid cavities within the spinal cord [15]. According to the MRI findings, patients were divided into positive findings group and negative findings group to identify the radiographic and clinical indicators of neural axis abnormalities between the two groups. Meanwhile, the infantile-age group and juvenile-age group were compared to determine the difference regarding prevalence of the neural axis abnormalities.

Anteroposterior and lateral standing full-spine radiographs were obtained at the first visit to record curve magnitude, curve type, location of curve apex, curve span and thoracic kyphosis (TK) measured from T5 to T12. Thoracic curve was defined with apex located from T2 to T11/T12 disc, thoracolumbar curve with apex from T12 to T12/L1 disc or L1, and lumbar curve with apex at L1/2 disc to L4 [16].

### Data analysis

Statistical analysis was performed using SPSS 16.0 statistical package (SPSS Inc., Chicago, IL). The student *t* test was used to evaluate the differences of continuous variables and the *chi-square* test was used to evaluate the difference of categorical variables. Fisher exact test was applied to detect the difference of the rate of intraspinal anomalies between the IIS and JIS group. Differences with a p value less than 0.05 were considered statistically significant.

## Results

A total of 504 patients were enrolled in this study, including 173 boys and 331 girls. The mean age of the patients at the first visit was 7.3 ± 2.8 years (range 16 months to 10 years). There were 288 main thoracic and 199 lumbar/thoracolumbar curves with an average curve magnitude of 30.4 ± 14.5° (range 20° to 64°).

**Fig. 1:**
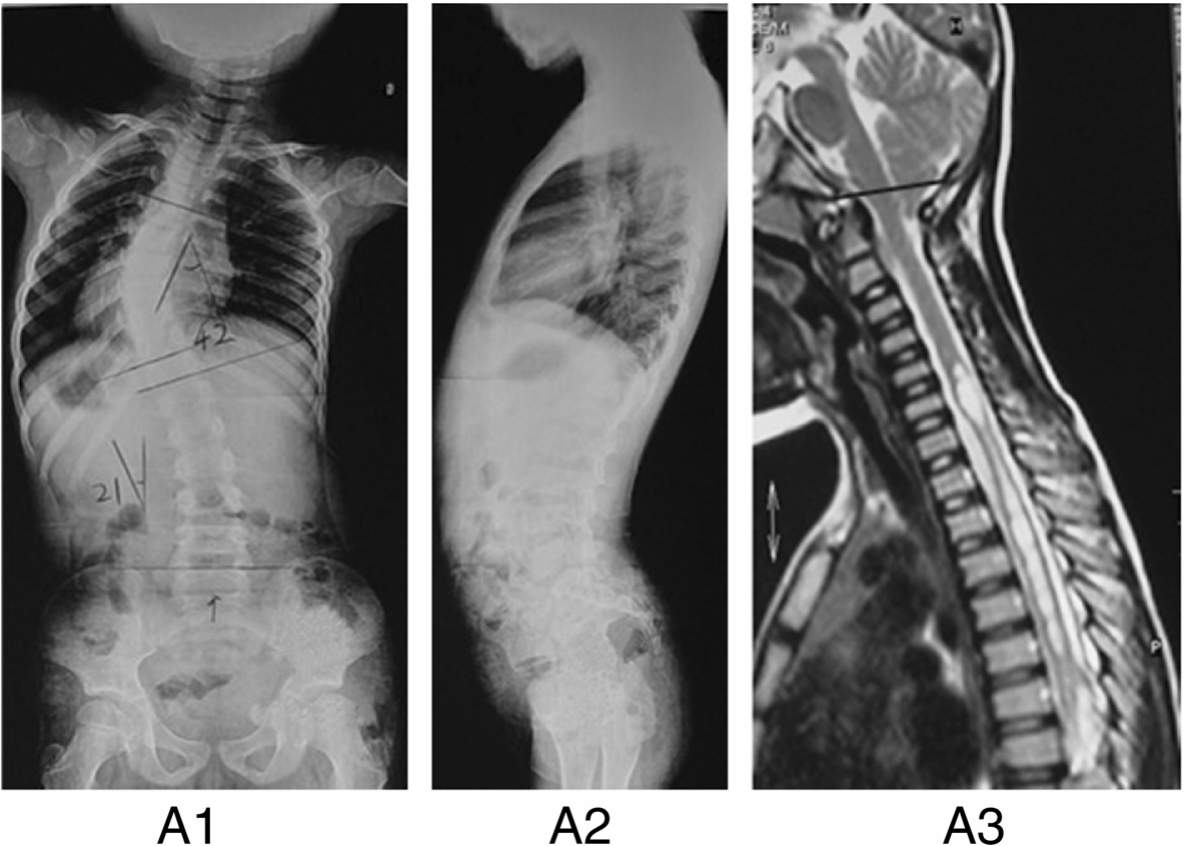
A 8-year-old boy diagnosed as presumed JIS with T5-T11 left-sided thoracic curve of 42° at first visit (A1-A2). He was screened by a full spinal MRI scan which demonstrated an Arnold-Chiari Type-I malformation combined with a moniliform syrinx extending from C5 –T5 seen on the T2 sagittal (A3). Then he underwent PFD (i.e. posterior fossa decompression)

94 (18.7 %) patients were found to have intraspinal neural axis abnormalities and all the positive findings of the spinal cord by MRI and their percentages were shown in Table 1. Totally Arnold-Chiari malformation with or without syringomyelia accounted for 64.8 % (61/94) of all these intraspinal neuro-axis abnormalities (Fig. 1) while diastematomyelia, tethered cord and intraspinal tumor were relatively uncommon. All the patients with neural axis abnormalities were referred to neurosurgical evaluation, treatment and follow-up accordingly.

**Table 1:** Description of neural abnormalities detected on preoperative MRI evaluation

Type of neural abnormality	Number of cases (%)
Isolated Arnold-Chiari malformation	43 (45.7 %)
Arnold-Chiari malformation combined with syringomyelia	18 (19.1 %)
Isolated syringomyelia	13 (13.8 %)
Tethered cord combined with diastematomyelia	6 (6.4 %)
Diastematomyelia	6 (6.4 %)
Tethered cord	4 (4.3 %)
Intrinsic spinal cord tumor	3 (3.2 %)
Syringomyelia combined with tethered cord and tumor	1 (1.1 %)
Total number	94

The results of comparison between the positive findings group and the negative findings group are summarized in Table 2. The percentage of male in group of abnormal MRI findings was significantly higher than that of the normal group (1: 1.04 vs. 1: 2.23, *p* = 0.021). Patients with a left thoracic curve were found to have higher rate of neural abnormalities (30.9 %, 21/68), when comparing to those with a right thoracic curve (40/220, 19.5 %) (*p* = 0.035). Likewise, six of the nine (66.7 %) patients with a right lumbar curve were found to have positive MRI findings, which was remarkably higher than that (14.1 %, 11/78) in the 78 patients with a left lumbar curve (*p* < 0.0001). As for the thoracolumbar curve, the patients with the right-sided curves had a higher rate of intraspinal anomalies than those with the left-sided curves but without statistical difference (p >0.05). There were no significant differences between the positive findings group and negative findings group with regard to age, curve magnitude, location of curve apex, TK and curve span. No significant difference was found regarding the incidence of intraspinal neuro-axis abnormalities between the IIS group and JIS group (15.5 % vs. 19.2 %, *p* > 0.05) (Table 2).

**Table 2:** Comparison between patients with and without neural abnormality on MRI screening examination

Descriptive data	Normal (*n* = 410)	Abnormal (*n* = 94)	*p* value
Gender			
Male	127	46	<0.0001
Female	283	48	
Average age at presentation (yrs.)	7.4 ± 2.3	7.2 ± 2.6	NS^a^
Diagnosis of scoliosis			
IIS	60	11	NS
JIS	350	83	
Main curve magnitude	29.6 ± 9.3°	35.1 ± 12.7°	NS^a^
Pattern of curve			
Left thoracic curve	47	21	
Right thoracic curve	180	40	0.025
Left lumbar curve	67	11	
Right lumbar curve	3	6	
Left thoracolumbar	77	4	<0.0001
Right thoracolumbar	27	4	NS^b^
Main curve apex location			
Thoracic	210	47	NS^b^
Thoracolumbar /lumbar	187	44	
Degree of thoracic kyphosis	17.5 ± 4.8°	18.9 ± 9.5°	NS^a^
Span of curve (levels)	6.4 ± 1.4	7.0 ± 1.2	NS^a^

^a^The student *t* test

^b^the chi-square test and otherwise the Fisher exact test was used

NS indicates no statistical significance

## Discussion

The intraspinal neural axis abnormalities commonly include Arnold-Chiari malformation [17], syringomyelia, tethered cord etc. It has been universally acknowledged that the intraspinal abnormalities can make the treatment of scoliosis a challenge, since it has been reported that the co-existing neural axis anomalies can potentially lead to a series of neural complications, such as paresthesia of the lower extremities, urinary retention, which may result from the intraoperative distraction of the spinal cord [18, 19]. Recent, some published studies have proved that the routine use of total spinal MRI scan for the presumed IIS and JIS could facilitate the identification of the intraspinal abnormalities and add to the understanding of the related indicators [1, 2, 4]. However, there is still a lack of consensus in the prevalence of intraspinal neuro-axis abnormalities with a wide range varying from 11.1 to 26.0 % [1–8] as shown in Table 3.

**Table 3:** Summary of incidences of neural axis abnormalities in presumed IIS and JIS by sample size and age at first visit

Study	Sample size	Age (yrs.)	Incidence (%)
Nakahara et al. [1]	53	≤11	13.2
Dobbs MB et al. [2]	46	≤3	21.7
Evans SC et al. [3]	31	4–12	26.0
Gupta et al. [4]	46	≤10	20.3
Inoue et al. [5]	73	<11	26.0
Koç T et al. [6]	72	≤7	11.1
Lewonowski et al. [7]	56	≤11	19.2
Pahys et al. [8]	54	<3	13.0
Current study	504	≤10	18.7

In the present study, patients with presumed IIS and JIS were found to have a prevalence of 18.7 % of the intraspinal neural axis abnormalities, which is close to the results of Dobbs MB et al. (21.7 %) [2], Gupta et al. (20.3 %) [4] and Lewonowski et al. (19.2 %) [7], while inconsistent with some reported results in other previous studies when comparing the widely varied rates in literature [1, 3, 5, 6, 8]. We believe that several methodological differences between the current and the previous studies, presumably including the inadequate sample and varied age span, may potentially compromise the validity of reported findings above. When setting the inclusion criteria of age, the subjects were investigated with the age at diagnosis of 7 years and younger by Koç T et al. [6], while we chose the patients younger or equal to 10 years of age, which followed the definition of IIS and JIS by the Scoliosis Research Society and found a prevalence of 18.7 %, which was higher than the prevalence of 11.1 % reported by Koç T et al. [6]. As for the sample size of the existing studies, the study with the largest sample to date was performed with only 73 patients by Inoue et al. [5], which may be insufficient to draw an accurate prevalence. While our study included a cohort of 504 patients, which we believe could facilitate a more valid conclusion. Furthermore, the Chiari malformation with or without syringomyelia accounted for half of the intraspinal neuro-axis abnormalities based on the results of the current study and much more attention should be poured into such abnormality [20]. As the patients with abnormal neuro-examinations were excluded in the protocol, the remaining abnormalities, such as diastematomyelia, tethered cord with some abnormal neurologic findings, were relatively rare in this study.

Since such a high prevalence of intraspinal neuro-axis abnormalities was found in our study, identifying the predictive factors was warranted. After comparing the radiological and clinical parameters between the positive findings group and negative findings group, the male sex, left thoracic curve and right lumbar curve were confirmed to be indicators for potentially-existing intraspinal abnormalities in patients with presumed IIS and JIS. As one significant feature of IS, the sex of female was more commonly seen than the male, whereas in scoliotic patients with neural axis abnormalities, the gender of male is reported to be more associated with neuro-axis deformities [4, 21]. Wu et al. [10] investigated the prevalence of neural axis abnormalities in asymptomatic patients aged from 7 to 24 years old with left thoracic scoliosis, showing that the ratio of male to female was over 2:1 (25 vs. 12) in 37 patients with neural axis abnormalities. However Pahys et al. [8] had different conclusion that the sex had no correlation with the intraspinal neuro-axis abnormalities in IIS. In present study, the ratio of male to female was 1: 1.04 in patients of positive findings group, which was significantly higher than that of negative findings group. Hence, it seems that the male sex could also be a strong indicator for neural axis abnormalities in presumed IIS and JIS patients and more concern should be raised on the male patients. In addition, the atypical curve patterns (namely, the left thoracic curve, right lumbar curve, and left thoracolumbar curve) have been proposed to be indicative for the intraspinal abnormalities in AIS patients [11, 22–24]. Similar with such studies, we found that the left thoracic and right lumbar curve were also strong indicators for early detection of neural axis abnormalities in IIS and JIS, while the right thoracolumbar curve failed to serve as an indicator in this study.

In previous studies, early-onset of scoliosis [25, 26], severe curves in immature individuals [4, 27], thoracic hyperkyphosis and long curve span [11] have been reported to be associated with neural axis abnormalities. Some authors noted that most AIS patients with intraspinal neural axis abnormalities may be identified based on such clinical and radiographic parameters [22, 28, 29], while our findings implied that those reported clinical and radiographic signs, including the age at first visit, main curve magnitude, main curve span, location of curve apex and TK may not indicate the intraspinal neuro-axis abnormalities for IIS and JIS patients. Thus, this could make it more challenging to detect the intraspinal neuro-axis abnormalities in patients with presumed IIS and JIS due to the less indicators confirmed in current study. A further study needs to be carried out to investigate the relationship between positive findings and related indicators. Collectively, the high prevalence of neuro-axis abnormalities could strongly support the importance of MRI for IIS and JIS at the first visit.

Some limitations exist in our study. Due to the inherent drawback of retrospective study, there was a lack of data on other reported predictors for neuro-axis abnormalities, including coronal imbalance, double main curve and backache. Second, subgroup analysis of differences of potential indicators between IIS and JIS was limited by the relatively small numbers in IIS patients. A further prospective study that includes more factors and larger sample size seems necessary for more conclusive results.

## Conclusions

Based on the largest group of patients in literature so far, the prevalence of neuro-axis abnormalities in patients with presumed IIS and JIS and a curve more than 20° was found to be 18.7 %. Our results therefore offer evidence that total spinal MRI evaluation is not over-requested but warranted for those presumed IS patients if aged less than 10 years, being male or having left thoracic or right lumbar curve.

### Ethics and consent to participate

The Drum Tower Hospital’s review board approved this retrospective study and waived the requirement for written informed consent for Dr. Zhu to review the radiographs, MR images and clinical data of enrolled patients.

### Consent to publish

Written informed consent was obtained from the patient’ parents for publication in this study and a copy of the written consent is available for review by the Editor of this journal.

### Availability of data and materials

The data and materials in current paper may be made available upon request through sending e-mail to first author.

## Abbreviations

IS: idiopathic scoliosis
IIS: infantile idiopathic scoliosis
JIS: juvenile idiopathic scoliosis
AIS: adolescent idiopathic scoliosis
PFD: posterior fossa decompression
